# Prenatal Exposure to Perfluoroalkyl Acids and Serum Testosterone Concentrations at 15 Years of Age in Female ALSPAC Study Participants

**DOI:** 10.1289/ehp.1408847

**Published:** 2015-06-02

**Authors:** Mildred Maisonet, Antonia M. Calafat, Michele Marcus, Jouni J.K. Jaakkola, Hany Lashen

**Affiliations:** 1 Department of Biostatistics and Epidemiology, College of Public Health, East Tennessee State University, Johnson City, Tennessee, USA; 2 Center for Environmental and Respiratory Health Research, Faculty of Medicine, University of Oulu, Oulu, Finland; 3 National Center for Environmental Health, Centers for Disease Control and Prevention, Atlanta, Georgia, USA; 4 Department of Epidemiology, Rollins School of Public Health, Emory University, Atlanta, Georgia, USA; 5 Faculty of Medicine, University of Sheffield, Sheffield, United Kingdom

## Abstract

**Background:**

Exposure to perfluorooctane sulfonic acid (PFOS) or to perfluorooctanoic acid (PFOA) increases mouse and human peroxisome proliferator–activated receptor alpha (PPARα) subtype activity, which influences lipid metabolism. Because cholesterol is the substrate from which testosterone is synthesized, exposure to these substances has the potential to alter testosterone concentrations.

**Objectives:**

We explored associations of total testosterone and sex hormone–binding globulin (SHBG) concentrations at age 15 years with prenatal exposures to PFOS, PFOA, perfluorohexane sulfonic acid (PFHxS), and perfluoronanoic acid (PFNA) in females.

**Methods:**

Prenatal concentrations of the perfluoroalkyl acids (PFAAs) were measured in serum collected from pregnant mothers at enrollment (1991–1992) in the Avon Longitudinal Study of Parents and Children (ALSPAC). The median gestational age when the maternal blood sample was obtained was 16 weeks (interquartile range, 11–28 weeks). Total testosterone and SHBG concentrations were measured in serum obtained from their daughters at 15 years of age. Associations between prenatal PFAAs concentrations and reproductive outcomes were estimated using linear regression models (*n* = 72).

**Results:**

Adjusted total testosterone concentrations were on average 0.18-nmol/L (95% CI: 0.01, 0.35) higher in daughters with prenatal PFOS in the upper concentration tertile compared with daughters with prenatal PFOS in the lower tertile. Adjusted total testosterone concentrations were also higher in daughters with prenatal concentrations of PFOA (β = 0.24; 95% CI: 0.05, 0.43) and PFHxS (β = 0.18; 95% CI: 0.00, 0.35) in the upper tertile compared with daughters with concentrations in the lower tertile. We did not find evidence of associations between PFNA and total testosterone or between any of the PFAAs and SHBG.

**Conclusions:**

Our findings were based on a small study sample and should be interpreted with caution. However, they suggest that prenatal exposure to some PFAAs may alter testosterone concentrations in females.

**Citation:**

Maisonet M, Calafat AM, Marcus M, Jaakkola JJ, Lashen H. 2015. Prenatal exposure to perfluoroalkyl acids and serum testosterone concentrations at 15 years of age in female ALSPAC study participants. Environ Health Perspect 123:1325–1330; http://dx.doi.org/10.1289/ehp.1408847

## Introduction

Perfluoroalkyl acids (PFAAs) constitute a class of synthetic chemicals whose water and oil repellency and surfactant properties have useful applications in a wide range of industrial and commercial products (Lindstrom et al. 2011). Some PFAAs are persistent and ubiquitous in the environment, and can be found in higher concentrations at higher levels in the food chain (Stockholm Convention 2014). Human exposure to PFAAs is common (Joensen et al. 2013; Kato et al. 2011). Detectable concentrations of some PFAAs in cord blood (Apelberg et al. 2007) and amniotic fluid from pregnant women (Stein et al. 2012) suggest human fetal exposure.

Effects of endocrine disruptors on reproduction may originate during the fetal stage (Crain et al. 2008), yet current knowledge on whether prenatal exposures to PFAAs can have an impact on human reproduction is limited to few studies. A longitudinal study of young adult females investigated associations of prenatal exposure to perfluorooctanoic acid (PFOA) and perfluorooctane sulfonic acid (PFOS) on menstrual characteristics, reproductive hormone levels, and number of follicles (Kristensen et al. 2013). In this study prenatal exposure to perfluorooctanoic acid (PFOA) was associated with delayed menarche attainment but not with other markers of reproductive function, whereas prenatal exposure to PFOS was not associated with any of the study outcomes (Kristensen et al. 2013). A nested case–control study conducted by our group explored the role of prenatal exposure to PFAAs on timing of menarche (Christensen et al. 2011). We did not find evidence of an association between prenatal exposure to the PFAAs tested and earlier menarche attainment. Last, a study of young adults explored whether prenatal PFOA or PFOS exposure was associated with semen quality, testicular volume, and reproductive hormone levels. Inverse associations of sperm concentration and total sperm count and positive associations of luteinizing and follicle-stimulating hormone with prenatal PFOA exposure were observed (Vested et al. 2013). To our knowledge, studies exploring effects of prenatal exposures to PFAAs on hormone concentrations in experimental animals have not been conducted.

Reproductive effects of many endocrine disruptors are thought to be mediated mainly by agonistic or antagonistic nuclear sex hormone receptor processes (Crain et al. 2008). It would appear, however, that PFAAs could impair human reproduction through other pathways. For instance, some PFAAs are known to increase mouse and human peroxisome proliferator–activated receptor alpha (PPARα) subtype activity (Takacs and Abbott 2007; Wolf et al. 2008), which influences lipid metabolism. Positive associations between exposure to PFAAs and lipids have been noted in adolescents and pregnant women from population-based cross-sectional epidemiologic reports (Geiger et al. 2014; Starling et al. 2014). Because cholesterol is the substrate from which reproductive hormones are synthesized (Burger 2002), exposure to PFAAs have the potential to alter concentrations of bioavailable circulating hormones. Alternatively, there is some evidence that endocrine disruptors, including PFOS, bind to sex hormone–binding globulin (SHBG) (Jones et al. 2003), and displacement of reproductive hormones from SHBG binding sites could alter bioavailability of circulating hormones (Déchaud et al. 1999).

High serum testosterone concentrations in females are a main feature of polycystic ovary syndrome (PCOS) that is associated with disruption of reproductive function (Azziz et al. 2009) as well as long-term sequelae such as metabolic disorders (Apridonidze et al. 2005) and possibly increased cardiovascular disease risk (Cibula et al. 2000). We report results of a study conducted to explore associations of prenatal exposures to PFOS, PFOA, perfluorohexane sulfonic acid (PFHxS), and perfluoronanoic acid (PFNA) with testosterone and SHBG serum concentrations at 15 years of age in females enrolled in the Avon Longitudinal Study of Parents and Children (ALSPAC). This study focused on female participants because data on prenatal PFAAs exposure in males enrolled in the ALSPAC are not yet available for analyses.

## Methods

*Source population.* The ALSPAC study enrolled pregnant women from three health districts of the old administrative county of Avon, United Kingdom, with an expected delivery date between April 1991 and December 1992. A total of 14,541 pregnant women, approximately 72% of the eligible source population, enrolled in the cohort during the 1990–1992 recruitment campaign. Details of recruitment methods are described elsewhere (Boyd et al. 2013).

*Study population.* We linked concentrations of PFAAs measured in enrolled pregnant women with their daughters’ serum concentrations of total testosterone and SHBG measured at 15 years of age using data from two separate investigations previously conducted on the ALSPAC. Maternal concentrations of PFAAs were measured in banked blood for a nested case–control study of prenatal exposures to PFAAs and menarche in daughters (Christensen et al. 2011). The median gestational age when maternal blood samples were obtained was 16 weeks [interquartile range (IQR), 11–28 weeks]. Total testosterone and SHBG concentrations were measured in serum samples collected from the 1,790 adolescent females who participated in the 15-years clinic visit open to all members of the ALSPAC cohort.

Cases in the nested case–control study consisted of 218 daughters who attained menarche at < 11.5 years and controls were a random sample of 230 daughters who attained menarche at ≥ 11.5 years of age (Christensen et al. 2011). We restricted the present study to daughters from the control sample (*n* = 230) because these are representative of the range of ages when menarche is most commonly attained. Not everyone who participated in the 15-years clinic visit had been a part of the case–control study; thus, after linkage of the 230 controls with the 1,790 clinic participants, only 72 females had prenatal concentrations of PFAAs and serum testosterone and SHBG concentrations at age 15 years available for the analyses.

*Covariates.* Covariates were selected *a priori*. Maternal covariates considered in the analyses were maternal smoking during pregnancy (yes, no); age at delivery (years); and prepregnancy body mass index (BMI; kilograms per meter squared) (Ernst et al. 2012; Sowers et al. 2001). Maternal educational level was also included in the set of maternal covariates as a measure of socioeconomic status (lowest: none/Certification of Secondary Education or Vocational; middle: Ordinary Level; highest: Advanced Level or University Degree). An Ordinary Level education is the qualification obtained at age 16 years when obligatory schooling ends, and an Advanced Level education is secondary or pre-university education certification. Daughters’ covariates considered in the analyses were serum SHBG concentrations at 15 years (nanomoles per liter), BMI at 15 years, age at menarche (< 12, 12–13, 14 years), and time of day the blood sample for testosterone testing was obtained (Ankarberg and Norjavaara 1999; Brand et al. 2011). Daughters’ BMI at age 15 years and age at menarche were also considered as covariates for the analyses for SHBG. There is a possibility that daughter’s BMI at 15 years might be affected by total testosterone and SHBG serum concentrations at 15 years or that daughter’s age at menarche might be affected by total testosterone serum concentrations. In such circumstances, use of BMI at 15 years or of age at menarche as covariates would be unnecessary. Data collection instruments and study variables have been described in detail elsewhere (ALSPAC 2014; Fraser et al. 2013).

*Laboratory analyses.* One total testosterone and one SHBG measure was made on serum samples obtained from daughters at age 15 years. About half of the blood samples were drawn between 0800 and 0900 hours and the other half between 1200 and 1500 hours. Total testosterone was measured using Agilent triple quadrupole 6410 liquid chromatography/mass spectrometry equipment with an electrospray ionization source operating in positive ion mode (Agilent Technologies, Wilmington, DE, USA). Multiple reaction monitoring was used to quantify total testosterone by using trideuterated testosterone (d3t-testosterone), with the following transitions: *m/z* 289.2-97 and 289.2-109 for testosterone and 292.2-97 and 292.2-109 for d3t-testosterone. The coefficients of variation of the method were 5.3, 1.6, and 1.2% for testosterone at 0.6, 6.6, and 27.7 nmol/L respectively. In an adult female population, the total testosterone reference range of 0.27–1.56 nmol/L has been suggested for measurements obtained by liquid chromatography/mass spectrometry (Kane et al. 2007). SHBG was measured using a Cobas Auto Analyzer (Roche Diagnostic, West Sussex, UK) and SHBG reagent using the manufacturer’s calibrators and quality control material.

PFOS, PFOA, PFHxS, and PFNA were measured in stored maternal sera collected during 1991–1992 at pregnancy. The median gestational age when samples were obtained was 15 weeks and the IQR was 10–28 weeks. Serum was analyzed using online phase extraction–high performance liquid chromatography–isotope dilution tandem mass spectrometry as described elsewhere (Kuklenyik et al. 2005). Limits of detection (LOD) were 0.2 ng/mL (PFOS), 0.1 ng/mL (PFOA, PFHxS), and 0.082 ng/mL (PFNA). A correction factor of 0.82 was applied to the PFNA concentrations to adjust for the purity of the analytic standards used [Centers for Disease Control and Prevention (CDC) 2013]. The precision of measurements, expressed as the relative standard deviation, was 8–13%, depending on the analyte. Prenatal concentrations of all PFAA analytes measured in the study group were above the LOD. Low-concentration (~ 2–9 ng/mL) and high-concentration (~ 6–25 ng/mL) quality control (QC) materials prepared with pooled serum were analyzed with standards, reagent blanks, and study samples. The concentrations of the QCs and blanks were evaluated using standard statistical probability rules.

*Statistical analyses.* Means of study outcomes by tertiles of prenatal PFAAs concentrations exhibited equal variances (data not shown). The null hypothesis for homogeneity of variances was tested separately for either total testosterone or SHBG concentrations with each prenatal PFAA using the Bartlett test, and none rejected at *p <* 0.05.

We used generalized additive models to detect whether associations between prenatal PFAA concentrations and the study outcomes exhibited departures from linearity (Hastie and Tibshirani 1987). We fitted these models with a smooth term for the continuous PFAAs for each prenatal PFAA and daughter’s outcomes combination separately. All smooth terms were fitted with 3 degrees of freedom. Chi-square tests from analyses of deviance comparing models with and without the smooth exposure term were performed, and no statistical departures from linearity were apparent at a *p* < 0.05 level of significance.

For analyses of prenatal continuous PFAAs, we estimated associations with total testosterone concentrations before and after exclusion of influential data points. We used studentized residuals (> 2.5 or < –2.5), leverage [>(2 number of predictor+2)/n], Cook’s distance (> 4/n), and visual observation of scatter plots as diagnostic tools to identify influential data points. After applying these criteria, two data points with corresponding values of PFOS of 66.4 and 69.2 ng/mL; two data points with corresponding values of PFOA of 13.8 and 14.6 ng/mL; three data points with corresponding values of PFHxS of 17.5, 49.8, and 54.1 ng/mL; and three data points with corresponding values of PFNA of 0.98, 1.07, and 1.15 ng/mL were excluded from respective models. It is important to underscore that influential data points identified using statistical methods do exhibit values of PFAAs concentrations observed in the human populations.

We report results from three separate linear regression models for the associations between the study outcomes and each PFAA: *a*) unadjusted model, *b*) fully adjusted, *c*) adjusted only by the covariates that were associated with the model’s respective PFAA at *p* < 0.20.

Human subject protection was assessed and approved by the ALSPAC Law and Ethics Committee and the Local Research Ethics Committees. Collection of pubertal data was also approved by the CDC Institutional Review Board. Human participants gave written informed consent before enrollment in the ALSPAC. Parental consent was obtained at the 15-years clinic visit because daughters were underage. Daughters could refuse any procedure requiring consent at any time during the clinic visit.

## Results

**Figure 1:**
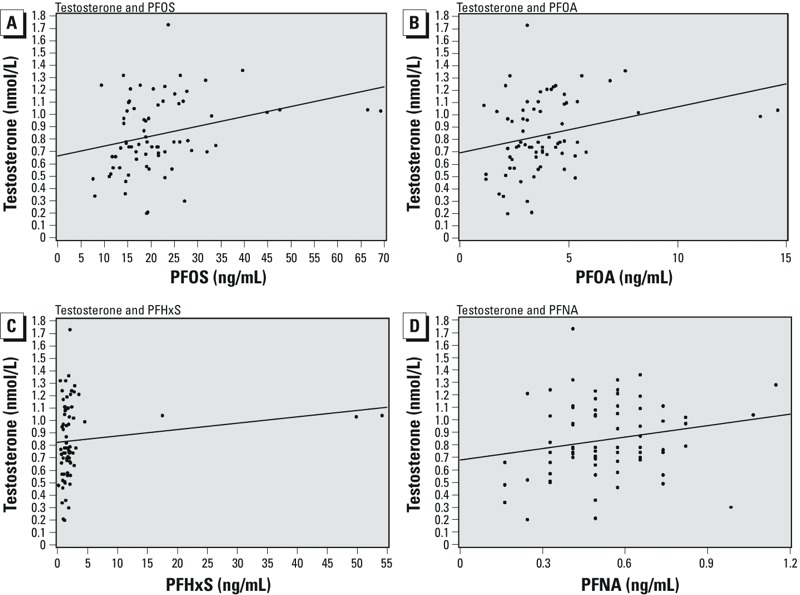
Scatter plots for total testosterone concentrations in 15-year-old daughters by prenatal PFOS (*A*), PFOA (*B*), PFHxS (*C*), and PFNA (*D*) serum concentrations with unadjusted linear regression line.

PFOS showed the highest median prenatal concentration (19.2 ng/mL) followed by PFOA (3.6 ng/mL), PFHxS (1.6 ng/mL), and PFNA (0.5 ng/mL) (Table 1). Spearman correlation coefficients suggest a moderate level of correlation between PFAAs: *r* values ranged between 0.47 and 0.65 (plots not shown). Median serum concentrations of total testosterone and SHBG in daughters at 15 years were 0.8 and 52.1 nmol/L, respectively (Table 1). The Pearson correlation coefficient between serum total testosterone and SHBG suggests a weak correlation (*r* = 0.11) between these two variables (plot not shown). The age range of the daughters in our study group was 15.0–15.9 years. All daughters had attained menarche and none used hormonal contraception. More than 50% of mothers reported having an advanced level or university degree education and delivering her daughter at ≥ 30 years of age. All of the mothers were of white race and had, on average, a normal prepregnancy weight (Table 2). Scatter plots for total testosterone concentrations in 15-year-old daughters by prenatal PFOS, PFOA, PFHxS, and PFNA serum concentrations with unadjusted linear regression line (Figure 1) suggest higher total testosterone serum concentrations with increasing values of prenatal PFAAs concentrations. There is also a suggestion of outlying values for PFOA, PFOS, and PFHxS.

**Table 1:** Distribution of PFAAs concentrations in serum from pregnant mothers and of outcomes in serum from daughters at 15 years of age.

Analyte	*n*	Minimum	25th	Median	75th	95th	Maximum
In maternal serum
PFOS^*a*^ (ng/mL)	72	7.6	15.1	19.2	25.0	44.9	69.2
PFOA^*a*^ (ng/mL)	72	1.1	2.7	3.6	4.7	7.6	14.6
PFHxS^*a*^ (ng/mL)	72	0.2	1.2	1.6	2.1	4.6	54.1
PFNA^*a*^ (ng/mL)	72	0.2	0.4	0.5	0.7	0.8	1.1
In daughters’ serum
Total testosterone (nmol/L)	72	0.2	0.7	0.8	1.1	1.3	1.7
Sex hormone-binding globulin (nmol/L)	72	7.9	37.6	52.1	72.73	110.3	135.3
25th, 75th, and 95th are percentiles. ^***a***^Limits of detection are for PFOS 0.2 ng/mL, for PFOA and PFHxS 0.1 ng/mL, and for PFNA 0.082 ng/mL.							

**Table 2:** Frequency distribution or means ± SDs for study covariates.

Covariates	*n* (%) or mean ± SD
Total	72 (100.0)
Maternal covariates
Smoking during first trimester
Yes	6 (8.3)
No	66 (91.7)
Educational level
Lowest	10 (13.9)
Middle	18 (25.0)
Highest	44 (61.1)
Race
White	72 (100)
Age at delivery (years)
< 25	8 (11.1)
25–30	28 (38.9)
≥ 30	36 (50.0)
Prepregnancy body mass index (kg/m^2^)	22.10 ± 3.27
Daughters’ covariates
Time of blood draw
0800–0900 hours	37 (51.4)
1200–1500 hours	35 (48.6)
Age at menarche (years)
< 12	4 (5.5)
12–13	57 (79.2)
14	11 (15.3)
BMI (kg/m^2^)	21.14 ± 2.57
Menarcheal	72 (100.0)
The mean age at menarche in the study group was 12.62 ± 0.90.	

Given the size of the study group, we created two adjusted models to determine associations between the outcomes and each prenatal PFAA separately. One adjusted model, which we refer to as the fully adjusted model, included all covariates identified *a priori*. The other, which we refer to as the parsimonious model, included only those covariates associated with the model’s corresponding PFAA at *p* < 0.20. In the fully adjusted linear regression model, total testosterone concentrations were on average 0.18-nmol/L [95% confidence interval (CI) = 0.01, 0.35] higher in the group of daughters with prenatal PFOS concentrations in the upper exposure tertile compared with the group of daughters with prenatal PFOS in the lower tertile (Table 3). This result was similar to that of the unadjusted PFOS model. In addition, none of the covariates were associated with prenatal PFOS at a chi-square *p*-value < 0.20; therefore, the parsimonious model is the same as the unadjusted model.

**Table 3:** Regression coefficients and 95% CIs from linear regression models for serum total testosterone concentrations (nmol/L) in 15-year-old daughters by tertiles and continuous prenatal PFAAs.

Concentration (ng/mL)	*n*	Mean ± SD	Unadjusted β (95% CI)	Fully adjusted β (95% CI)^*a*^	Parsimonious β (95% CI)^*b*^
PFOS
≤ 15.8	24	0.76 ± 0.29	Reference	Reference	Reference
15.9–22.6	24	0.79 ± 0.26	0.03 (–0.14, 0.20)	0.10 (–0.07, 0.28)	0.03 (–0.14, 0.20)
> 22.6	24	0.97 ± 0.32	0.20 (0.03, 0.37)	0.18 (0.01, 0.35)	0.20 (0.03, 0.37)
Per 1 ng/mL			0.008 (0.002, 0.014)	0.006 (–0.001, 0.012)	0.008 (0.002, 0.014)
PFOA
≤ 2.9	24	0.74 ± 0.29	Reference	Reference	Reference
2.9–4.1	24	0.82 ± 0.32	0.07 (–0.09, 0.24)	0.15 (–0.02, 0.32)	0.10 (–0.07, 0.27)
> 4.1	24	0.96 ± 0.26	0.22 (0.05, 0.39)	0.24 (0.05, 0.43)	0.18 (0.01, 0.36)
Per 1 ng/mL			0.037 (0.006, 0.068)	0.033 (–0.001, 0.066)	0.030 (–0.002, 0.061)
PFHxS
≤ 1.2	24	0.74 ± 0.31	Reference	Reference	Reference
1.3–1.9	24	0.83 ± 0.28	0.10 (–0.07, 0.26)	0.18 (0.00, 0.37)	0.14 (–0.05, 0.33)
> 1.9	24	0.95 ± 0.29	0.21 (0.04, 0.38)	0.18 (0.00, 0.35)	0.20 (0.02, 0.38)
Per 1 ng/mL			0.005 (–0.003, 0.014)	0.003 (–0.006, 0.012)	0.005 (–0.004, 0.014)
PFNA
≤ 0.4	26	0.81 ± 0.34	Reference	Reference	Reference
0.5–0.6	25	0.84 ± 0.30	0.04 (–0.13, 0.21)	0.08 (–0.10, 0.25)	0.07 (–0.11, 0.24)
> 0.6	21	0.88 ± 0.26	0.07 (–0.11, 0.25)	0.05 (–0.14, 0.24)	0.10 (–0.08, 0.28)
Per 1 ng/mL			0.249 (–0.045, 0.543)	0.224 (–0.089, 0.536)	0.292 (–0.003, 0.586)
^***a***^Each PFAA was modeled separately and adjusted by SHBG concentration, maternal education, maternal age at delivery, maternal prepregnancy BMI, maternal smoking during pregnancy, time of day daughter’s blood sample was obtained, daughter’s age at menarche, and daughter's BMI at 15 years. ^***b***^Each PFAA was modeled separately and adjusted only by those covariates associated with the corresponding PFAA at *p* < 0.20. Models were adjusted by PFOS, none included; PFOA, maternal education; PFHxS, maternal smoking during pregnancy, maternal age at delivery, and maternal prepregnancy BMI; PFNA, time of day daughter’s blood sample was obtained.					

Similar patterns were observed for PFOA (β = 0.24; 95% CI: 0.05, 0.43) and PFHxS (β = 0.18; 95% CI: 0.00, 0.35), where total testosterone levels in fully adjusted models were on average higher in the group of daughters with prenatal exposure in the upper tertile compared with the group in the lower tertile (Table 3). The parsimonious model for PFOA, which was adjusted only by maternal education, showed similar results (β = 0.18; 95% CI: 0.01, 0.36) to those of the fully adjusted model. The parsimonious model for PFHxS, adjusted by maternal smoking during pregnancy, maternal age at delivery, and maternal prepregnancy BMI, also showed similar results (β = 0.20; 95% CI: 0.02, 0.38) to those of the fully adjusted PFHxS model. In the fully adjusted model, the difference in average total testosterone concentrations in daughters with prenatal PFNA concentrations in the upper tertile compared with daughters with prenatal PFNA in the lower tertile was smaller (β = 0.05; 95% CI: –0.14, 0.24) than those observed for other PFAAs. The PFNA parsimonious model (β = 0.10; 95% CI: –0.08, 0.28), which was adjusted only for time of day when daughter’s blood sample was obtained, showed a somewhat higher average total testosterone in daughters with prenatal PFNA in the upper tertile compared with daughters with prenatal PFNA in the lower tertile.

In separate regression models using the same adjustment approach described above (Table 3), we also explored associations of total testosterone concentrations in daughters with continuous prenatal PFAAs. In fully adjusted models, differences in total testosterone concentrations per nanogram per milliliter increase were rather similar for PFOS (β = 0.006; 95% CI: 0.001, 0.012), PFOA (β = 0.033; 95% CI: 0.001, 0.066), and PFHxS (β = 0.003; 95% CI: –0.006, 0.012), compared with differences in total testosterone concentrations per nanogram per milliliter increase in PFNA (β = 0.224; 95% CI: –0.089, 0.536). Overall, results from fully adjusted models and from parsimonious models were similar. After exclusions of influential data points, the difference in total testosterone concentration per unit increase in fully adjusted models for PFOS was 0.009 (95% CI: 0.000, 0.018); PFOA was 0.056 (95% CI: 0.000, 0.112); PFHxS was 0.083 (95% CI: –0.016, 0.182); and PFNA was 0.281 (95% CI: –0.011, 0.671). Compared with results from models before exclusion of influential data points, regression coefficients were slightly larger.

In the fully adjusted models, SHBG concentrations were on average higher in daughters with prenatal PFOS (β = 3.46; 95% CI: –12.06, 18.98), PFOA (β = 5.02; 95% CI: –13.07, 23.11), and PFNA (β = 7.91; 95% CI: –8.69, 24.52) concentrations in the upper tertile compared with daughters with prenatal concentrations in the lower tertile (Table 4). In contrast, SHBG concentrations were on average lower in daughters with prenatal PFOS (β = –2.86; 95% CI: –18.80, 13.09) and PFNA (β = –4.53; 95% CI: –19.96, 10.90) in the middle tertile compared with the lower tertile in respective fully adjusted models. For PFHxS (β = –5.3; 95% CI: –21.61, 11.00), SHBG concentrations were on average lower in the group of girls with prenatal concentrations in the upper tertile compared with those in the lower tertile. Parsimonious models showed rather similar results to those of their respective fully adjusted models.

**Table 4:** Regression coefficients and 95% CIs from linear regression models for serum SHBG concentrations (nmol/L) in 15-year-old daughters by tertiles of prenatal PFAAs.

Concentration (ng/mL)	*n*	Mean ± SD	Unadjusted β (95% CI)	Fully adjusted β (95% CI)^*a*^	Parsimonious β (95% CI)^*b*^
PFOS
≤ 15.8	24	56.71 ± 30.42	Reference	Reference	Reference
15.9–22.6	24	54.31 ± 25.15	–2.40 (–18.01, 13.20)	–2.86 (–18.80, 13.09)	–2.40 (–18.01, 13.20)
> 22.6	24	59.29 ± 25.40	2.58 (–13.02, 18.19)	3.46 (–12.06, 18.98)	2.58 (–13.02, 18.19)
PFOA
≤ 2.9	24	55.77 ± 33.50	Reference	Reference	Reference
2.9–4.1	24	60.24 ± 26.07	4.47 (–11.11, 20.05)	0.32 (–15.97, 16.61)	3.83 (–11.54, 19.19)
> 4.1	24	54.30 ± 19.85	–1.47 (–17.06, 14.11)	5.02 (–13.07, 23.11)	5.45 (–10.50, 21.40)
PFHxS
≤ 1.2	24	57.87 ± 33.09	Reference	Reference	Reference
1.3–1.9	24	60.61 ± 24.19	2.74 (–12.76, 18.25)	–2.22 (–19.62, 15.19)	0.04 (–17.17, 17.24)
> 1.9	24	51.82 ± 22.23	–6.05 (–21.55, 9.45)	–5.31 (–21.61, 11.00)	–6.40 (–23.01, 10.21)
PFNA
≤ 0.4	26	57.90 ± 27.50	Reference	Reference	Reference
0.5–0.6	25	51.75 ± 25.59	–6.15 (–21.17, 8.88)	–4.53 (–19.96, 10.90)	–5.15 (–20.61, 10.32)
> 0.6	21	61.34 ± 27.62	3.44 (–12.29, 19.18)	7.91 (–8.69, 24.52)	4.52 (–11.70, 20.73)
^***a***^Each PFAA was modeled separately and adjusted by maternal education, maternal age at delivery, maternal prepregnancy BMI, maternal smoking during pregnancy, time of day daughter’s blood sample was obtained, daughter’s age at menarche, and daughter BMI at 15 years. ^***b***^Each PFAA was modeled separately and adjusted only by those covariates associated with the corresponding PFAA at *p* < 0.20. Models were adjusted by PFOS, none included; PFOA, maternal education; PFHxS, maternal smoking during pregnancy, maternal age at delivery, and maternal prepregnancy BMI; PFNA, time of day daughter’s blood sample was obtained.					

## Discussion

We explored the influence of prenatal exposure to PFOS, PFOA, PFHxS, and PFNA on testosterone and SHBG serum concentrations at 15 years of age in females. In our study of 72 ALSPAC daughters, prenatal exposure to PFOA, PFOS, and PFHxS were associated with higher total testosterone concentrations. To our knowledge, there is only one previous published study where associations of prenatal PFOS and PFOA with total testosterone and SHBG concentrations were determined (Kristensen et al. 2013). In this study, total testosterone and SHBG were obtained at 20 years of age in a sample of 75 female users of non-hormone contraceptives who were enrolled in a population-based Danish cohort with magnitude of exposure similar to that of our study group. Kristensen et al. (2013) constructed linear regression models using continuous PFOS and PFOA exposure and natural log–transformed study outcomes, adjusted by mother’s smoking and income, and daughter’s smoking, BMI, luteinizing and follicule-stimulating hormones, and estradiol. For total testosterone, the regression coefficient for the association with PFOS (β = 0.009) in the study by Kristensen et al. (2013) appeared to be of similar magnitude to that of our study, though it exhibited wider confidence intervals. The regression coefficient for the association with PFOA (β = –0.003) was closer to the null than the one in our study, and it also exhibited wider confidence intervals. For SHBG, the regression coefficients for the association with PFOS (β = 0.006) and PFOA (β = 0.011) in the study by Kristensen et al. (2013) were close to the null compared with those in our study. Variations in covariates used in the adjusted regression analyses and use of natural log–transformed outcomes could explain the differences between the results of Kristensen et al. (2013) and our study.

Testosterone has been reported to exhibit both circadian and menstrual cycle variation (Ankarberg and Norjavaara 1999; Bui et al. 2013). These represent sources of random error that could bias measures of association. We used time of day the daughters’ blood sample was drawn to explore circadian variation and included this variable in the multivariate regression analyses. We did not have information on the time of the menstrual cycle when blood samples were obtained, so it remains a potential source of variation in our study.

Testosterone circulates in the bloodstream mostly bound to SHBG (Davison and Davis 2003). SHBG was not associated with prenatal PFAA exposure in our study. Other studies did not find associations between SHBG and either prenatal or cross-sectional exposure to PFAAs in male or female adolescents (Joensen et al. 2013; Kristensen et al. 2013; Vested et al. 2013). Although PFAAs appear to have an affinity for SHBG binding sites (Jones et al. 2003), it is not known whether exposure to PFAAs could actually alter circulating SHBG concentrations.

We did not find clear evidence of an association of prenatal exposure to PFNA with circulating testosterone concentrations. Coefficients were close to the null for the tertile estimates, but the coefficients for the continuous exposure were substantially larger than for the other PFAAs, though much less precise. We found that PFNA exhibited lower median serum concentration and narrower IQR than the other analytes tested. In a representative sample of the U.S. population, PFNA levels went up since the phaseout of PFOS in the early 2000s (Kato et al. 2011), possibly because it is used as a PFOS substitute. In our study, serum samples were collected from pregnant women before the phaseout of PFOS; thus PFNA exposure levels are low compared with more recent exposure levels seen in the United States (Kato et al. 2011) and elsewhere (Joensen et al. 2013).

Daughter’s BMI at 15 years and age at menarche were introduced in the fully adjusted regression models. If daughter’s BMI at 15 years and age at menarche are actually descendent of the study outcomes, their use as a covariate in the model is unnecessary. Neither daughter’s BMI at 15 years nor age at menarche remained in the models adjusted only by covariates that were associated with the PFAA at *p* < 0.20. In addition to not having an impact on precision, their exclusion from the models did not appear to meaningfully change the magnitude of the regression coefficients, so it does not affect bias.

The mode of action through which prenatal exposures to the PFAAs could alter testosterone concentrations remains to be explained, but may involve several pathways. One mode of action could involve alterations in cholesterol metabolism through PPARα activation. Alterations in the expression of genes associated with lipid transport, lipoprotein metabolism, and cholesterol biosynthesis in fetal livers of mice prenatally exposed to PFOA, consistent with PPARα activation, suggest opportunities of fetal programming of PFOA (Rosen et al. 2007). Another mode of action could involve activation of the PPARγ subtype. PPARγ is believed to regulate energy homeostasis and appears to be involved in fertility (Froment et al. 2006). PPARγ is expressed in adipose tissue, which is where conversion of about half of the body’s circulating testosterone occurs. Neither PFOS nor PFOA appears to increase mouse or human liver PPARγ activity in a cell assay study (Takacs and Abbott 2007). To our knowledge, it is unclear whether prenatal exposure to PFAA could alter adipose tissue PPARγ activity. Exposure to the PFAAs was not associated with menarche timing in our study population (Christensen et al. 2011), but it was inversely associated with birth weight (Maisonet et al. 2012), and birth weight could be regulated by PPAR activation. Last, other modes of action could include increased levels of gonadotropins (Vested et al. 2013) or reduced conversion to estrogens.

Associations between PFAA exposure and delayed menarche attainment observed in longitudinal and cross-sectional epidemiologic studies (Kristensen et al. 2013; Lopez-Espinosa et al. 2011) and between PFAA exposure and increased time to pregnancy and irregular menstrual cycles (Fei et al. 2009) in cross-sectional studies suggest that alterations in testosterone levels may be a relevant mode of action.

Our study is of an exploratory nature; we answered the study questions using a small group of enrolled cohort members whose exposure and outcome data had been collected for other purposes. Medians and IQRs of exposure and outcome measures in our study group were generally consistent with the distribution of these variables in their source samples (data not shown). Because we excluded girls with early menarche attainment from the analyses, selection bias could occur if prenatal PFAAs exposure influences age at menarche. In previous analysis, however, we did not find clear evidence on an association between prenatal PFAAs exposure and earlier menarche attainment (Christensen et al. 2011). Analysis was conducted in a sample of females because data on prenatal exposure to PFAAs are not currently available for males enrolled in the ALSPAC cohort; nonetheless, the exploration of associations between prenatal exposures to PFAAs and hormones levels in males is of scientific relevance.

Results of our study suggest a possibility for fetal programming by prenatal PFAA exposure. Besides the fetal stage, there are other postnatal, developmental stages when hormone production or signaling processes could be vulnerable to effects of endocrine disruptors. In addition, human exposure patterns to PFAAs are changing on a global scale as a result of changes in regulatory policies. To better understand how endocrine disruptors alter human reproduction, it would be valuable to design epidemiologic studies with the capacity to address multiple vulnerability periods and time-varying exposures.
